# Mortality Audit in the Head and Neck Surgery Ward: A Retrospective Study in a Tertiary Care Hospital of Pakistan

**DOI:** 10.7759/cureus.58869

**Published:** 2024-04-23

**Authors:** Saleh Khurshied, Saad A Khan, Shana Sagheer, Hassan Arslan, Muhammad H Rafique, Nawal Khurshid, Hammad Ahmed

**Affiliations:** 1 Otolaryngology - Head and Neck Surgery, Pakistan Institute of Medical Sciences, Islamabad, PAK; 2 Internal Medicine, Aga Khan University Hospital, Karachi, PAK; 3 Medicine and Surgery, Rawal Institute of Health Sciences, Rawalpindi, PAK; 4 Pediatrics, Fauji Foundation Hospital, Rawalpindi, PAK

**Keywords:** death, ward, audit, mortality, malignancy

## Abstract

Background

Mortality audit is important for healthcare workers, but this data is lacking in developing countries. It helps to provide material about the cause of death, mortality rate, age, and gender. In a surgical department, such information can help identify key public health challenges that are contributing to morbidity and mortality, and this information can help healthcare workers better tackle those pathologies and focus on their prevention and treatment.

Materials and methods

A retrospective study was conducted at the Department of ENT - Head and Neck Surgery, Pakistan Institute of Medical Sciences Hospital, Islamabad. Five-year data was collected from the mortality register of the ward from January 2019 to December 2023, including the age, gender, surgical diagnosis, course of hospital stay, and cause of death. The collected data was statistically analyzed and presented in the form of tables and figures.

Results

A total of 53 deaths in 3890 admissions were found on record, with an overall mortality rate of 1.4%. The median age of participants was 61.5 years, with a preponderance of the male gender (n=34; 64.2%). The most common cause of death was head and neck malignancy (n=39; 73.6%), followed by head and neck abscesses (n=9; 17%). The least common cause of death was diphtheria (n=2; 3.8%).

Conclusion

Death was more common in old-age patients, with more prevalence in the male population. The most common cause of mortality was head and neck malignancy. The total death count almost remained constant through the years.

## Introduction

By auditing, we mean to check and regulate the quality of medical practice [1]. Mortality statistics of admitted patients reproduce the reasons for major illnesses and care provided. The World Health Organization (WHO) has made criteria for medical certification that list different diseases that happen in a sequential order, resulting in death along with other associated conditions that are not directly associated with the cause of death [2]. Statistics showing the prevalence of disease and morbidity and mortality are important for healthcare workers, but this data is lacking in developing countries. This data provides information regarding the cause of death, mortality rate, age of deceased, and sex [3]. By finding mortality patterns, we can improve surgical care and the effectiveness of treatment options, which will eventually benefit the community [4]. Mortality audits help in the implementation of better practices, which help to improve care and facilities provided to patients [5]. This study was done to assess death patterns in admitted patients of the ENT/Head and Neck Surgery ward, which will help in the improvement of the provision of healthcare by highlighting the causes of death. The objective of this study was to determine the demographics and causes of mortality in admitted patients of the ENT ward in a tertiary care hospital.

## Materials and methods

This retrospective descriptive observational study was carried out at the Department of ENT - Head and Neck Surgery, Pakistan Institute of Medical Sciences (PIMS) Hospital, Islamabad, after obtaining formal written consent from the head of the department to use the mortality data from the ward mortality register. Data was collected from the mortality register of the ward from January 2019 to December 2023. The mortality register contains information regarding the age, gender, surgical diagnosis, and clinical reason for death of the patients who had died in the ward. Co-morbid conditions that did not contribute to the mortality, such as diabetes mellitus, hypertension, dyslipidemia, etc., were excluded. 

Inclusion criteria included all patients, irrespective of age, gender, or socio-economic background, who died while admitted to the ENT ward or died within one month of admission [6]. This data included all patients who died during their admission to the ENT ward, irrespective of whether surgery was done or not. Patients who died due to head and neck pathologies in the emergency department, at home (where the last admission was greater than one month ago), or while admitted to another specialty's services (including medical oncology and internal medicine) were excluded.

The collected data was entered and statistically analyzed using Statistical Package for Social Services v25 (IBM Inc., Armonk, New York) for Windows. Descriptive data was presented in the form of frequencies and percentages which were displayed in the form of tables and charts. The primary variable under study was the clinical cause of death, while age and gender were secondary variables.

The information that could identify patients was hidden while collecting data to maintain patient confidentiality. No direct interaction with any patient or their families took place.

## Results

**Figure 1:**
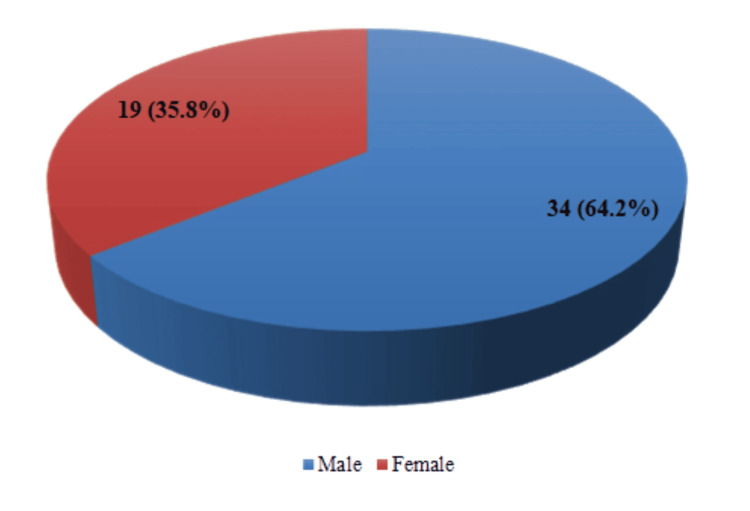
Gender-specific mortality Number of deaths in male and female patients over the past five years (January 2019 to December 2023) along with percentage proportion of total deaths for each gender in brackets

A total of 3890 patients were admitted to the ward between January 2019 and December 2023 (2763 male patients and 1127 female patients), and 53 deaths were noted during this duration. Out of these 53 deaths, 34 (64.2%) were male and 19 (35.8%) were female deaths, as shown in Figure 1.

Table 1 demonstrates the number of admissions in each year and the mortality rate among admissions per individual year. Table 2 shows the year-wise mortality rate while the distribution of mortality in various age groups is shown in Table 3.

**Table 1:** Number of admissions and deaths in individual years Number of admissions (% of total admissions) = Total number of admissions in the corresponding year with percentage proportion of total admitted patients corresponding to that year in brackets Number of deaths (% of total deaths) = Total number of deaths in the corresponding year with percentage proportion of total deaths corresponding to that year in brackets Percentage of admitted patients dying = Percentage of admitted patients that passed away during the corresponding year.

Year	Number of admissions (% of total admissions)	Number of deaths (% of total deaths)	Mortality rate
2019	854 (22.0%)	11 (20.7%)	1.3%
2020	733 (18.8%)	12 (22.6%)	1.6%
2021	444 (11.4%)	9 (17.0%)	2.0%
2022	872 (22.4%)	11 (20.7%)	1.3%
2023	987 (25.4%)	10 (18.9%)	1.0%
Total	3890 (100%)	53 (100%)	1.4%

**Table 2:** Year-wise mortality distribution N = Total number of deaths in each corresponding year (%) = Percentage proportion of total deaths for each corresponding year in brackets

Year	No of deaths N (%)
2019	11 (20.7)
2020	12 (22.6)
2021	9 (17)
2022	11 (20.7)
2023	10 (18.9)
Total	53 (100)

**Table 3:** Age specific mortality Age range (years) = The range of ages, in years, of the patients who died during the study period Number of deaths (% of total mortality) = The number of deaths in that age group along with percentage proportion of total deaths corresponding to that age group in brackets Deaths in males (% of total mortality) = The number of deaths of male patients in that age group along with percentage proportion of total deaths corresponding to male patients in that age group in brackets Deaths in female (% of total mortality) = The number of deaths of female patients in that age group along with percentage proportion of total deaths corresponding to female patients in that age group in brackets

Age range (years)	Number of deaths (% of total mortality)	Deaths in males (% of total mortality)	Deaths in females (% of total mortality)
1-10	1 (1.89)	1 (1.89)	0 (0)
11-20	1 (1.89)	0 (0)	1 (1.89)
21-30	2 (3.77)	2 (3.77)	0 (0)
31-40	5 (9.43)	4 (7.55)	1 (1.89)
41–50	8 (15.09)	5 (9.43)	3 (5.66)
51-60	13 (24.53)	8 (15.09)	5 (9.43)
61-70	21 (39.62)	12 (22.64)	9 (16.98)
71-80	2 (3.77)	2 (3.77)	0 (0)
81-90	0 (0)	0 (0)	0 (0)
Total	53 (100)	34 (64.2)	19 (35.8)

The age of the deceased patients ranged from nine years to 81 years with a median age of 61.5 years.

**Figure 2:**
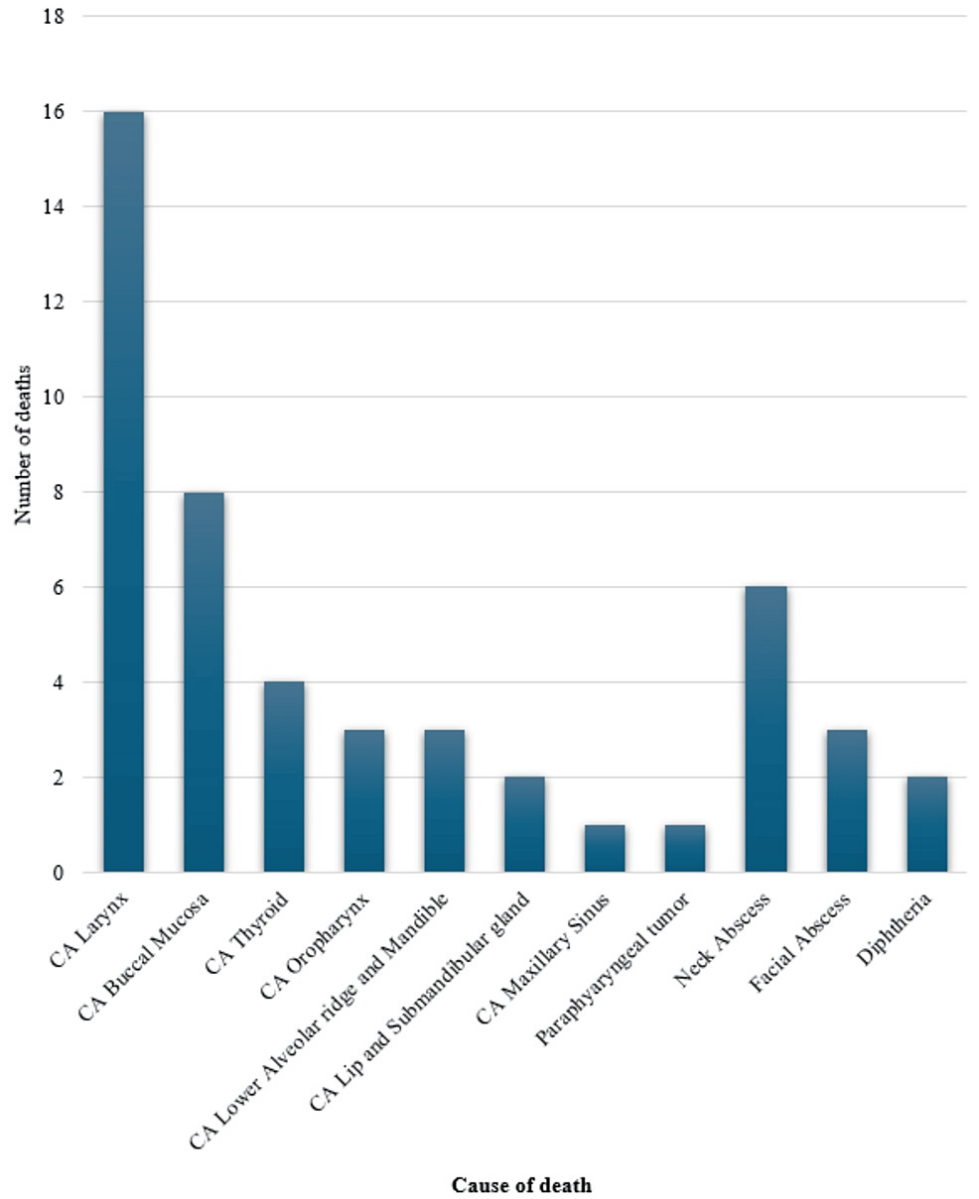
Clinical conditions causing mortalities in ENT ward y-axis (number of deaths) = Number of deaths corresponding to the disease x-axis (cause of death) = Common diseases leading to death in the ward CA = Cancer

The most common cause of mortality in ENT wards as per our audit was malignancy (n=39; 73.6%), consisting of 16 cases of laryngeal cancer (30%); four cases of thyroid cancer (7.5%), eight cases of buccal mucosa malignancy (15.1%), three cases of lower alveolar ridge and mandible cancer (5.7%), three cases of oropharyngeal cancer (5.7%), two cases of lip and submandibular gland malignancy (3.8%), one case of maxillary sinus tumor (1.9%) and one case of parapharyngeal tumor (1.9%). Malignancy was followed by abscesses (n=9; 17.0%), which included six cases of neck abscesses (11.3%) and three cases of facial abscesses (5.7%). Other causes include diphtheria causing two (3.8%) deaths. Figure 2 shows the clinical conditions causing mortalities in our study

## Discussion

About 57 million deaths are reported every year worldwide, three-fourths (76.7%) of which occur in developing countries, among which Southeast Asia contributes about 22% of the total. Continuous review of mortality is thus necessary for improving the provision of the healthcare system [7].

The majority of studies done worldwide mainly focus on mortality in patients presenting in ENT emergency; thus, data on the mortality of admitted patients is lacking. The chief reason for death in our study was malignancy (75%), followed by abscesses (16.7%) and diphtheria (8.3%). A discussion among the faculty and residents of the ward was done to ascertain the likely causes of the mortality patterns seen in the study. High mortality in malignancy-related cases seemed to be due to the presentation of patients at an advanced stage, primarily due to neglect by patients and families either in diagnosis or treatment [8]. Most carcinoma cases are usually admitted for palliative care due to the irresectable nature of the disease or distant metastasis post-chemo radiotherapy. Poor socioeconomic status and poor hygiene are often associated with neck abscesses, which are common in our settings. Patients with deep neck space abscesses were also noted with late presentation and significant extension, mainly due to reduced awareness or absence of appropriate care, which eventually led to mortality. [9,10]

In their study, Kumar et al. [11] found the median age of patients who died in the ENT ward to be 40.60 years, while in our study, it was 61.50 years. They also found that mortality due to malignancy in their study was 32.75%, but in our study, it was higher (i.e., 73.6%) mainly because they also included deaths in emergencies as well. Chukuezi et al. [12] did their study in surgical wards and found male mortality to be higher than female, which was consistent with our results. Ayoade et al. [6] in their research found mortality due to carcinoma to be 29.7%, while in our research, it was 75%, but they also included ENT emergencies, which were excluded from our study. The common prevalence of non-malignant, acute conditions in younger patients in the emergency department (as opposed to malignancies, which are chronic conditions and often present in the ward) can explain these differences.

Saha et al. [13], in their audit, found laryngeal cancer to be the cause of 47% of deaths. Osuji et al. [14] found that male mortality was higher than female mortality, and laryngeal tumors were the most common cause of mortality (47.8%); these findings were similar to our study.

Around one to two percent of admitted patients died each year, which remained constant throughout the study period. This was similar to the studies by Kumar et al. (0.9%), Saha et al. (2.1%) and Osuji et al. (1.8%) [11, 13, 14]. However, when data from other surgical departments was taken into account as well, the mortality rate rose significantly as traumatic injuries and GI complications (conditions not present in the head and neck ward) contributed to a significant number of deaths [8, 12].

Public and healthcare professionals' awareness regarding early recognition of signs of malignancy can aid in early diagnoses and starting of treatment, which can help decrease the mortality in admitted patients. Thus, regular mortality audits and taking preventative actions can help decrease the adverse effects by changing clinical practices [15].

We presented the data from the last five years, and our results correspond to the limited research done on this subject. Our study had a few limitations; data from only the last five years was used, and data was collected from a single hospital. No details of the clinical course of the disease, the extent of abscesses formation and the staging and grading of tumors was available in the retrospective data; hence the impact of extent of disease at presentation on mortality could not be ascertained. We recommend further long-term and multicentric research on this subject so that results could be more generalized.

## Conclusions

Death was more common in old-age patients, with a higher prevalence in male patients. The total death count and annual mortality rate remained constant throughout the years. The pattern of death showed that the main reason for death was head and neck malignancies, possibly due to a delay in diagnosis and treatment. This highlights the importance of educating the public on early signs of head and neck malignancies as well as control of established risk factors of the condition (i.e., smoking, alcohol and betel nut consumption, oral hygiene, etc.). Control of these risk factors can also help limit the incidence of abscesses, the second leading cause of death in our study. 
